# Novel AXL-specific inhibitor ameliorates kidney dysfunction through the inhibition of epithelial-to-mesenchymal transition of renal tubular cells

**DOI:** 10.1371/journal.pone.0232055

**Published:** 2020-04-23

**Authors:** Atsuo Kurata, Yukako Tachibana, Tadakatsu Takahashi, Naoshi Horiba

**Affiliations:** Research Division, Chugai Pharmaceutical Co., Ltd., Gotemba, Shizuoka, Japan; National Cancer Institute, UNITED STATES

## Abstract

Chronic kidney diseases affect more than 800 million people globally and remain a high unmet need. Various therapeutic targets are currently under evaluation in pre-clinical and clinical studies. Because the growth arrest specific gene 6 (Gas6)/AXL pathway has been implicated in the pathogenesis of kidney diseases, we generated a novel selective and potent AXL inhibitor, CH5451098, and we evaluated its efficacy and elucidated its mechanism in an NEP25 mouse model that follows the clinical course of glomerular nephritis. In this model, CH5451098 significantly ameliorated the excretion of urinary albumin and elevation of serum creatinine. Additionally, it also inhibited tubulointerstitial fibrosis and tubular damage. To elucidate the mechanism behind these changes, we analyzed the effect of CH5451098 against transforming growth factor β1 (TGFβ1) and Gas6, which is a ligand of AXL receptor, in NRK-52E renal tubular epithelial cells. CH5451098 inhibited epithelial-to-mesenchymal transition (EMT) caused by the synergistic effects of TGFβ1 and Gas6 in NRK-52E cells. This inhibition was also observed in NEP25 mice. Taken together, these results suggest that CH5451098 could ameliorate kidney dysfunction in glomerular nephritis by inhibiting EMT in tubular cells. These results reveal that AXL strongly contributes to the disease progression of glomerular nephritis.

## Introduction

Chronic kidney diseases are one of the common causes of cardiac disorders, which subsequently leads to more than 1 million deaths annually worldwide [1]. Current therapies are insufficient and outcomes following immunosuppression or antihypertensive drugs are unsatisfactory [2, 3]. There is a great demand for light to be shed on mechanisms underlying the progression of kidney diseases. Elucidation of these mechanisms, in particular those involving molecule-specific approaches, offers hope for the development of effective drugs.

AXL, belonging to TAM family (AXL, Mer and, Tyro3) of receptor tyrosine kinases, binds with the strongest affinity the ligand Gas6, which is a common ligand for TAM receptors [4–7]. The Gas6/AXL pathway regulates cell proliferation, adhesion, migration, and inflammation, but its most well-known function is in cell growth. Thus, many therapeutics targeting AXL are currently under development in the oncology field [8, 9].

In rodents and humans, kidney AXL and Gas6 are expressed in glomerular mesangial cells and tubular cells [10–12]. Their expression is far more abundant in the kidneys of IgA nephritis and lupus nephritis patients than in healthy volunteers [10]. The extracellular domain of AXL is reported to be shed from the cell surface by proteases and deposited in blood [13, 14]. Serum concentrations of this soluble AXL and the Gas6 ligand increase in patients with class 3 or 4 lupus nephritis and are well correlated with the disease activity index [15–19]. Moreover, soluble AXL decreases in patients responding to conventional therapy, but remains stable in non-responders [20]. Although this suggests that AXL and Gas6 strongly contribute to disease progression of glomerular nephritis, the precise mechanism of AXL in kidney diseases remains unknown.

Activation of the AXL receptor by Gas6 in mesangial cells has been shown to promote glomerular damage in several animal models [10, 21–24], but many of these are limited, focusing only on glomerulosclerosis or tubulointerstitial fibrosis [25]. We employed NEP25 mouse model for exploring the effects of therapeutic interventions of an AXL inhibitor because the model can follow the clinical course of glomerular nephritis followed by tubulointerstitial fibrosis within three weeks [26]. This information is a key to develop a future therapy that will satisfy the high unmet medical needs arising from kidney diseases like glomerular nephritis.

In this study, we identified CH5451098 as a novel and potent AXL inhibitor showing greater selectivity for AXL over other kinases. We evaluated the effect of AXL inhibition on kidney diseases in NEP25 mice using CH5451098. In addition, to precisely elucidate the underlying mechanism, we examined its inhibitory potency against epithelial-to-mesenchymal transition (EMT) in NRK-52E tubular epithelial cells.

## Materials and methods

### Reagents

**Fig 1:**
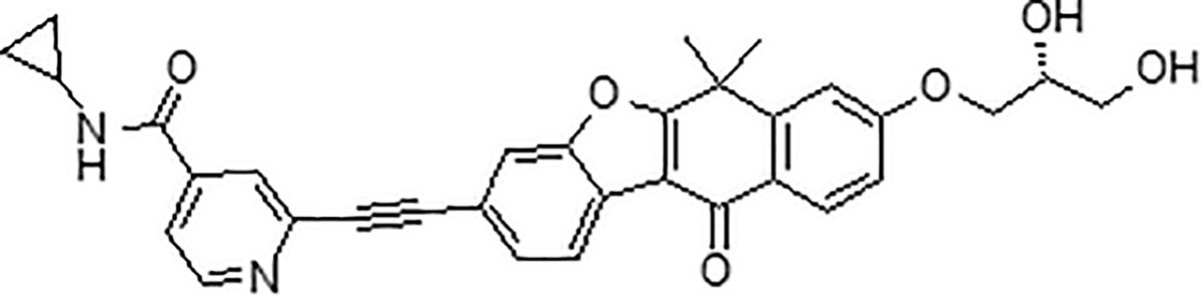
Chemical structure of CH5451098.

CH5451098 (N-cyclopropyl-2-[2-[8-[(2R)-2,3-dihydroxypropoxy]-6,6-dimethyl-11-oxo-naphtho[2,3-b]benzofuran-3-yl]ethynyl]pyridine-4-carboxamide), a selective inhibitor of AXL, was synthesized at Chugai Pharmaceutical Co. Ltd. (Fig 1). The following recombinant proteins were used for the kinase assay: AXL, Mer, Tyro3, Flt3, JAK2, EGFR, HER2, KIT, PDGFRβ, FGFR2, KDR, MET, ALK, EPHA2, EPHB2, FAK, SRC, ABL, ERK1, PKA, AKT1, PKACα, PKACβ1, AurA, CHK1, CHK2, MNK1, CDK1, and p38α from Carna Biosciences (Kobe, Japan), and Raf1 from Thermo Fisher Scientific (Waltham, MA). Recombinant mouse TGFβ1 (mTGFβ1) and recombinant mouse Gas6 (mGas6) were purchased from R&D systems. All other chemicals were of the highest purity available. The following primary antibodies were used for this study: anti-beta catenin (8480), anti-zinc finger E-box-binding homeobox 1 (Zeb1) (3396), anti-glyceraldehyde-3-phosphate dehydrogenase (GAPDH) (8884) antibody from Cell Signaling Technology (Danvers, MA). As a secondary antibody, this study used Anti-rabbit IgG (7074) from Cell Signaling Technology.

### Protein kinase assay

Protein kinases were purchased from Carna Biosciences or Millipore. The inhibitory activity against each kinase was evaluated as described previously [27].

### Animals

NEP25 mice, heterozygous podocyte-specific hCD25 transgenic mice, were bred in Tokai University and kindly provided by Matsusaka et al. [26]. All mice were kept in well-controlled animal housing with free access to water and pellet food. Animal procedures and protocols were in accordance with the Guidelines for the Care and Use of Laboratory Animals at Chugai Pharmaceutical Co. Ltd. and approved by the Institutional Animal Care and Use Committee.

### In vivo experimental design

To induce disease, NEP25 mice were injected with 0.8 μg/kg of the immunotoxin LMB2 (provided from National Institutes of Health, USA) via the tail vein, except in the normal control group (n = 4). CH5451098 powder was suspended in a 0.5% carboxymethyl cellulose solution to prepare 1.0 mg/mL. Mice were divided into 2 groups and were orally administered vehicle (disease group, n = 10) or CH5451098 10 mg/kg once daily for 21 days (CH5451098 group, n = 10) from one day before LMB2 injection. The dose of CH5451098 was set high enough to inhibit AXL kinase activity throughout the day. Mice not treated with LMB2 were used as normal control.

Blood and urine samples were collected on day 7, 14, or, 21 after LMB2 injection. Blood samples were collected from the abdominal portion of the vena cava under isoflurane anesthesia and were centrifuged to obtain serum samples. Urine samples were collected for 24 hours using a metabolic cage. The mice were euthanized by exsanguination under isoflurane anesthesia at day 22 after LMB2 injection. Kidney tissues were dissected for pathology (formalin-fixed or methyl carnoy-fixed) and kidney cortex samples were collected and snap frozen in liquid nitrogen.

### Biochemical analysis

Serum urea nitrogen (BUN), creatinine (sCre) and urinary albumin (uAlb), N-Acetyl-beta-D Glucosaminidase (NAG), and creatinine were measured using an automatic analyzer (Hitachi 7100 Autoanlayzer, Hitachi Co., Ltd. Tokyo, Japan).

### Gene expression analysis

RNA samples were extracted from frozen mouse kidney cortex or NRK-52E cells using an RNaesy Mini kit (QIAGEN, Hilden, Germany). cDNA were synthesized from RNA using a reverse transcription kit (Roche applied science, Penzberg, Germany). Expression levels were measured by a LightCycler 480 (Roche Applied science) using TaqMan probe/primer sets for each gene (Thermo Fisher Scientific).

### Western blotting

The kidney cortex was lysed with a buffer containing cell lysis buffer (Cell Signaling Technology), EDTA, and Halt Protease & Phosphatase inhibitor single-use cocktail (Thermo Fisher Scientific). Samples with an equivalent amount of protein were analyzed by SDS-PAGE. Proteins were electroblotted onto PVDF membranes (Bio-Rad laboratories) and membranes were blocked with PVDF Blocking Reagent (TOYOBO, Tokyo, Japan) then incubated sequentially with primary and secondary antibodies. Protein detection was performed using SuperSignal West Dura Extended Duration Substrate (Thermo Fisher Scientific) and LAS-4000 (Fujifilm, Tokyo, Japan).

### Collagen analysis

To assess collagen content in the kidney, we measured 4-Hydroxyproline (OH-Pro) content by a colorimetric method. Briefly, the chopped renal specimens were hydrolyzed overnight in 6N HCl at 95°C. After filtrating the boiled samples, aliquots of hydroxylate were measured with a colorimetric method followed the procedure (BioVision, Zurich, Switzerland). The amount of OH-Pro was determined by comparison with a standard curve prepared from known concentrations of the OH-Pro reagent. The renal OH-Pro content was normalized by tissue dry weight.

### Pathological analysis

Kidney tissues were fixed with 10% neutral buffered formalin solution or methyl carnoy’s fixative and embedded in paraffin. The paraffin blocks were cut in 4 μm and stained with Sirius red, Masson’s Trichrome or, periodic acid-Schiff. Kidney interstitial fibrosis was analyzed on Masson’s Trichrome-stained sections for each mouse using the following semi-quantitative criteria as described previously [28, 29]: grade 0: normal kidney, grade 1: damage to up to 25% of the cortex, grade 2: damage to 26 to 50% of the cortex, and grade 3: damage to >50% of the cortex.

### Cells

NRK-52E cell line derived from rat renal tubular epithelial cells was purchased from ATCC (Manassas, VA) and cultured in Dulbecco’s modified eagle’s medium with high glucose media supplemented with 5% FBS at 37°C under 5% CO 2 and 95% air.

### In vitro experimental design

NRK-52E cells were seeded onto a collagen-coated plate and were cultured over-night. After reaching subconfluence, cells were cultured in serum restricted medium (0.5% FBS) for 6 hours. Then the cells were stimulated with recombinant mTGFβ1 (2 ng/mL) and/or recombinant mGas6 (500 ng/mL) followed by treatment with or without CH5451098 (1 nmol/L) for 15 minutes. The cells were lysed with RLT buffer (Qiagen) after 2 hours incubation with stimulants and mRNA was purified using RNeasy mini kit (Qiagen).

### Statistical analysis

Statistical analysis was performed by Wilcoxon rank sum test for pathological score, Dunnett’s t-test for in vitro analysis or Student’s t-test for all other analyses using Prism GraphPad7 (GraphPad Software, La Jolla, CA). A one-way ANOVA was performed before Dunnett’s t-test. Statistical significance was set at p<0.05, 001, and 0.001.

## Results

### Activity and selectivity of CH5451098

**Fig 2:**
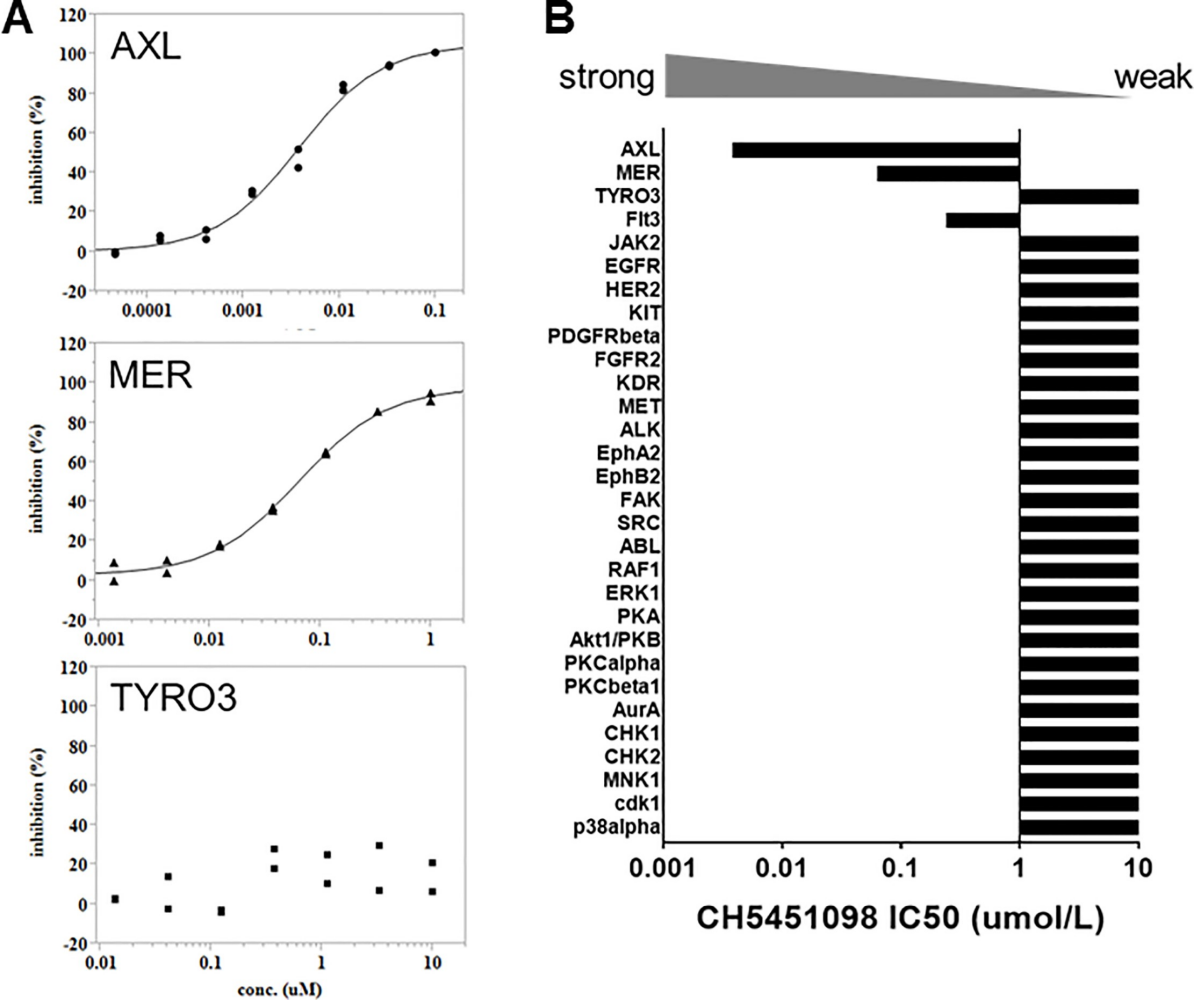
In vitro kinase inhibitory activity of CH5451098. A, dose-dependent inhibitory activity of CH5451098 in TAM family receptors (AXL, MER and TYRO3). Recombinant protein of AXL, MER, and TYRO3 were treated with CH5451098 for 1.5 hours at the indicated doses. Enzyme inhibitory activity was calculated by the formula (1-T/C) x 100 (%), where T represents the measured value of the wells with a compound and C represents that of wells without a compound (n = 2). EC50 and Top (Emax) values were estimated by fitting the relationships between the concentrations of compounds (x) and the inhibitory activity (y) according to the Emax model by using nonlinear regression analysis of JMP® (Ver. 11.2.1, SAS Institute Japan). B, The IC50 values of CH5451098 in 30 kinase inhibitory assays. IC50 values were shown as mean (n = 2).

We generated the novel AXL inhibitor CH5451098 and evaluated its inhibitory activity. The inhibitory activity of CH5451098 against AXL is shown in Fig 2A. CH5451098 dose-dependently inhibited AXL kinase activity and its IC50 was calculated as 3.8 nmol/L. We also evaluated selectivity against other kinases. CH5451098 displayed the strongest inhibitory activity towards AXL among 30 kinases including the TAM family (Tyro3, AXL and, Mer) of receptor tyrosine kinases (RTKs) (Fig 2B).

### In vivo efficacy of CH5451098

**Fig 3:**
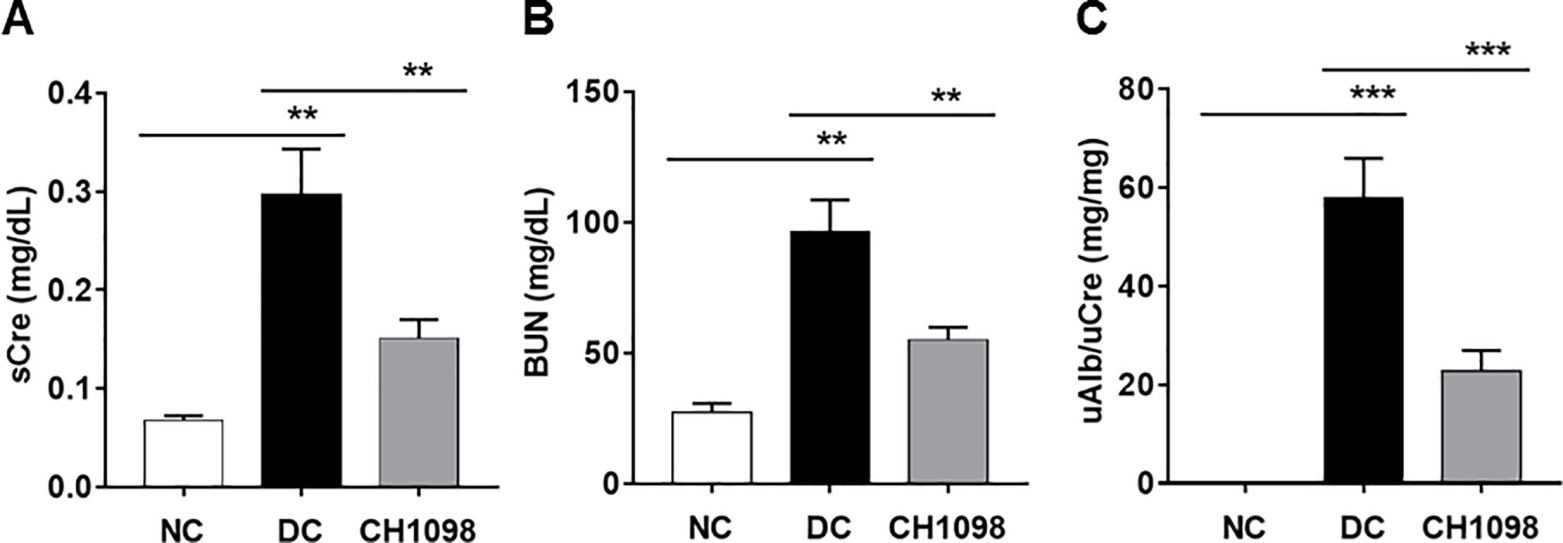
Effects of CH5451098 on kidney function in the NEP25 mouse model. CH5451098 10 mg/kg or vehicle was administered on 21 consecutive days starting one day before LMB2 injection. Blood was collected at 24 hours and urine was collected from 0 to 24 hours after final administration of CH5451098 or vehicle. sCre (A), BUN (B), and uAlb (C) were measured. Concentration of uAlb was normalized against creatinine in urine. Data are expressed as mean ± SE: n = 4 in NC, n = 7 in DC, n = 10 in CH5451098 treatment, *p<0.05, **p<0.01, ***p<0.001, significant difference between NC and DC, or DC and CH5451098 treatment with Student’s t-test. NC, normal control; DC, disease control; CH1098, CH5451098.

NEP25 mice gradually developed kidney dysfunction as measured by sCre level (S1 Fig). Renal AXL mRNA expression level increased in accordance with the disease progression (S2 Fig). On the other hand, mRNA expression of other TAM family members in the kidney significantly increased or decreased, but these changes were not so much compared with the change of AXL mRNA (S3 Fig). To investigate its therapeutic potency, the AXL inhibitor CH5451098 was administered to NEP25 mice daily starting one day before disease induction. Serum creatinine and BUN significantly increased in disease control compared with normal control, and those increases were significantly ameliorated by treatment with CH5451098 (sCre: 0.30 ± 0.046 versus 0.15 ± 0.019 mg/dL, p<0.01. Fig 3A, BUN: 97 ± 12 versus 55 ± 5.0 mg/dL, p<0.01. Fig 3B). CH5451098 also significantly decreased uAlb excretion compared with disease control (Fig 3C).

**Fig 4:**
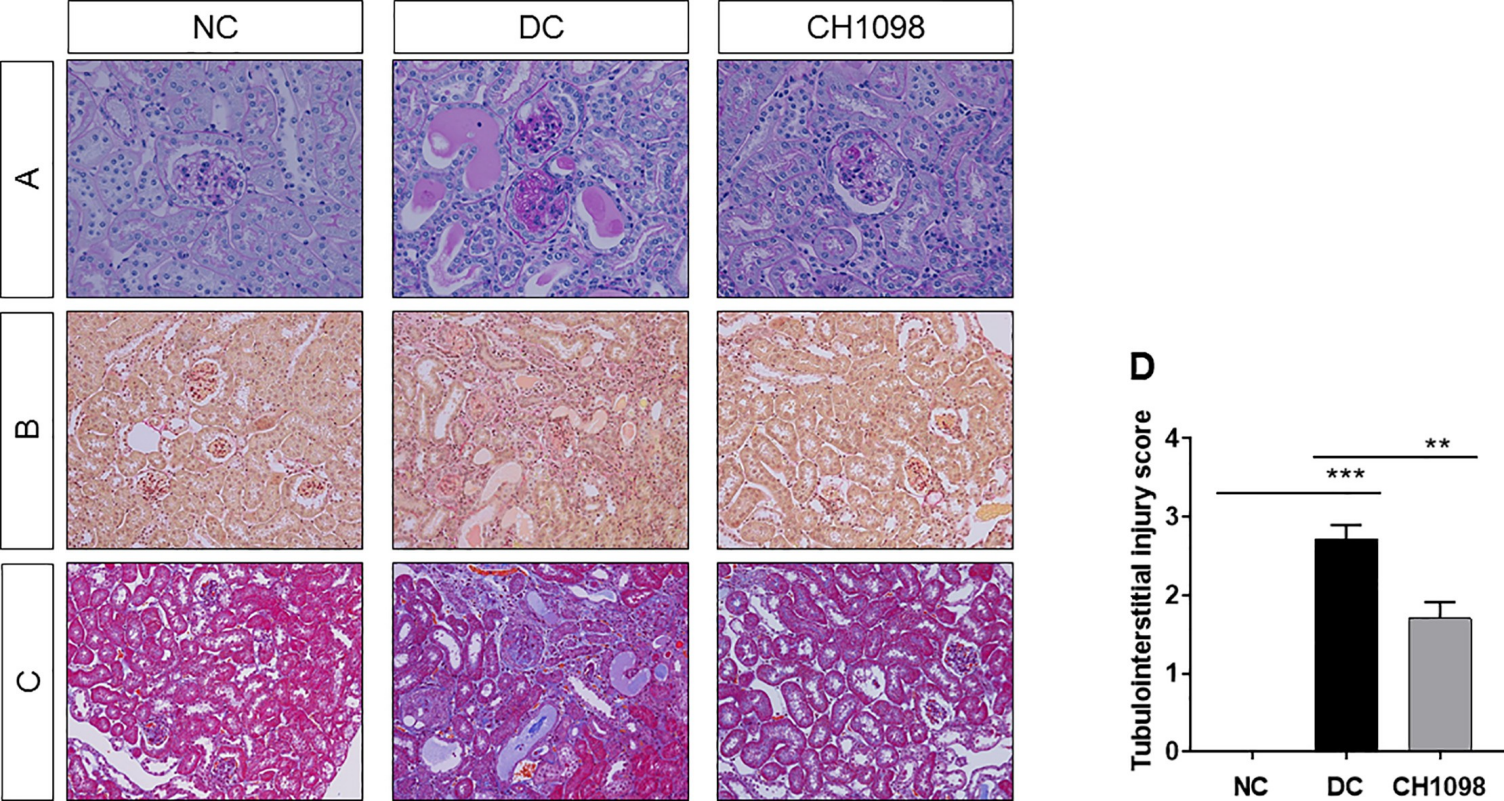
Effects of CH5451098 on glomerulosclerosis and tubulointerstitial fibrosis in NEP25 mouse model. CH5451098 10 mg/kg or vehicle was administered on 21 consecutive days starting one day before LMB2 injection. Images of periodic acid-Schiff stain, x40 (A), Sirius red stain, x20 (B), Masson’s Trichrome stain, x20 (C) from NC, DC, and CH1098. Semi-quantification of Masson’s Trichrome for tubulointerstitial injury score (D). Data are expressed as means ± SE: n = 4 in NC, n = 7 in DC, n = 10 in CH5451098 treatment *p<0.05, **p<0.01, ***p<0.001, significant difference between NC and DC, or DC and CH5451098 treatment with Wilcoxon rank sum test. NC, normal control; DC, disease control; CH1098, CH5451098.

**Fig 5:**
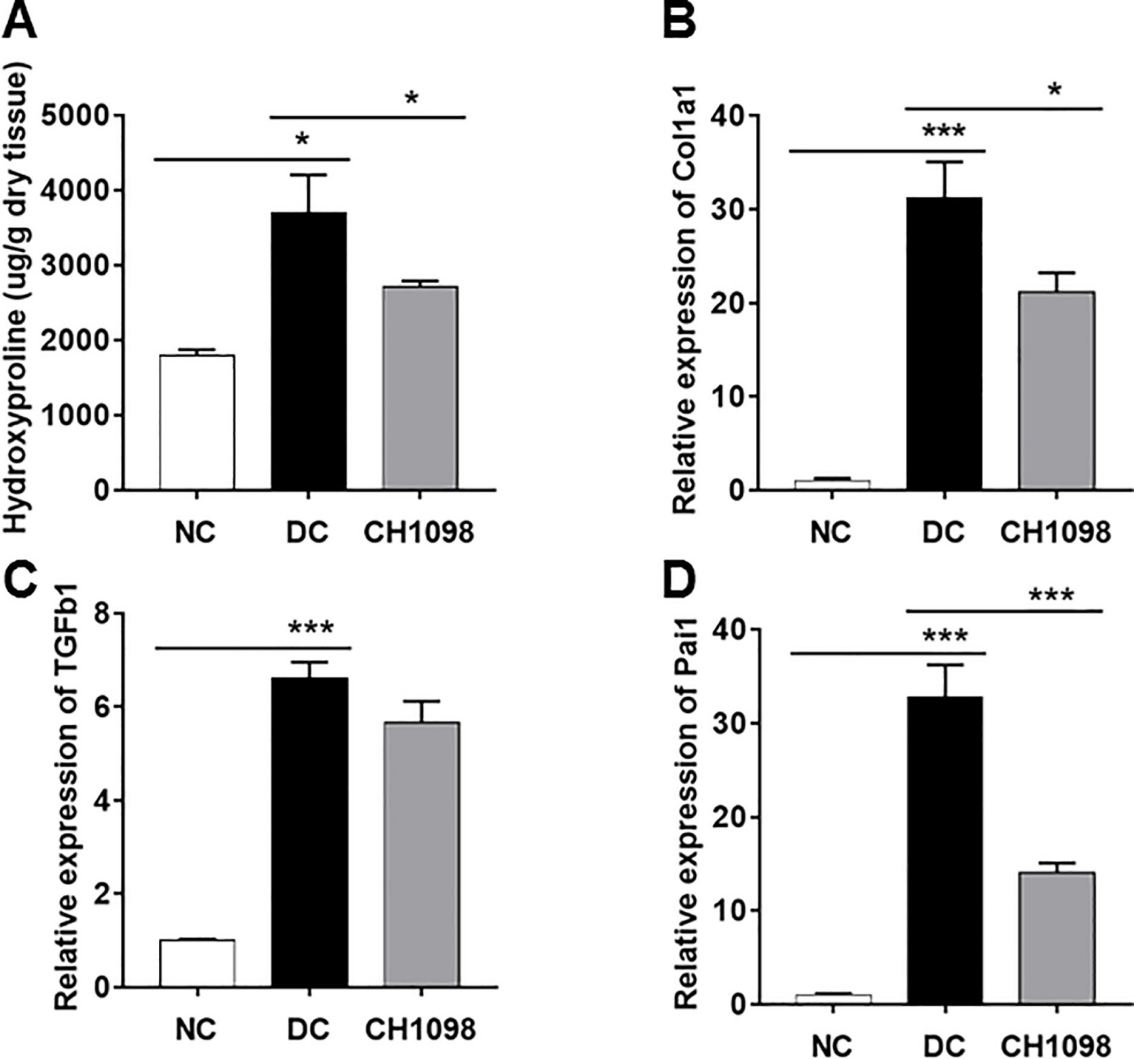
Effects of CH5451098 on kidney fibrosis in NEP25 mouse model. CH5451098 10 mg/kg or vehicle was administered on 21 consecutive days from one day before LMB2 injection. Kidney cortex was collected at 24 hours after final administration of CH5451098 or vehicle and measured for Hydroxyproline content (A), Col1A1 mRNA (B), TGFβ1 mRNA (C), or Pai1 mRNA (D). mRNA expression levels of each were expressed as the fold changes of NC following normalization by mitochondrial ribosomal protein L19 (Mrpl19) mRNA. Data are expressed as mean ± SE: n = 4 in NC, n = 7 in DC, n = 10 in CH5451098 treatment *p<0.05, **p<0.01, ***p<0.001. significant difference between NC and DC, or DC and CH5451098 treatment with Student’s t-test. NC, normal control; DC, disease control; CH1098, CH5451098.

The potency of the AXL inhibitor was also confirmed by the pathological findings (Fig 4). The destruction of glomerular structure exhibited in disease control was ameliorated by treatment with CH5451098. CH5451098 ameliorated not only glomerulus damage, but also sustained normal tubular structure (Fig 4A). To characterize the pharmacological mechanism of CH5451098 in the NEP25 model we analyzed anti-fibrotic properties in kidney. In Sirius red and Masson’s Thrichrome staining analysis, abundance of stained connective tissue observed in the disease control group was inhibited by CH5451098 (Fig 4B and 4C). To analyze the effects of CH5451098 on tubulointerstitial fibrosis, we evaluated tubular damage on Masson’s trichrome-stained sections (Fig 4D). CH5451098 significantly suppressed tubulointerstitial fibrosis according to semi-quantitative analysis. In the disease control group, the abundant connective tissue was observed in the area surrounding dilated tubules. Based on the histochemical analysis, we measured OH-Pro, which is found in collagen and elastin in mammals and is known to be correlated with the amount of extracellular matrix protein [30]. OH-Pro was elevated in disease control and the elevated OH-Pro was suppressed with CH5451098 treatment (Fig 5A). The anti-fibrotic effect was also supported by the decrease of kidney collagen 1a1 (Col1a1) mRNA, which is reported to have high mRNA expression in fibrotic tissue (Fig 5B) [31]. CH5451098 significantly reduced the expression of Col1a1 mRNA compared to disease control. TGFβ1 is understood as a master regulator of fibrosis, and the induction of EMT is one its mechanisms. Plasminogen activator inhibitor-1 (Pai1) mRNA, which is thought to be PD marker of TGFβ signaling, was significantly inhibited by CH5451098 treatment compared to disease control, even though TGFβ1 mRNA expression showed only a partial reduction (Fig 5C and 5D). These results suggest that CH5451098 blocked the TGFβ pathway.

**Fig 6:**
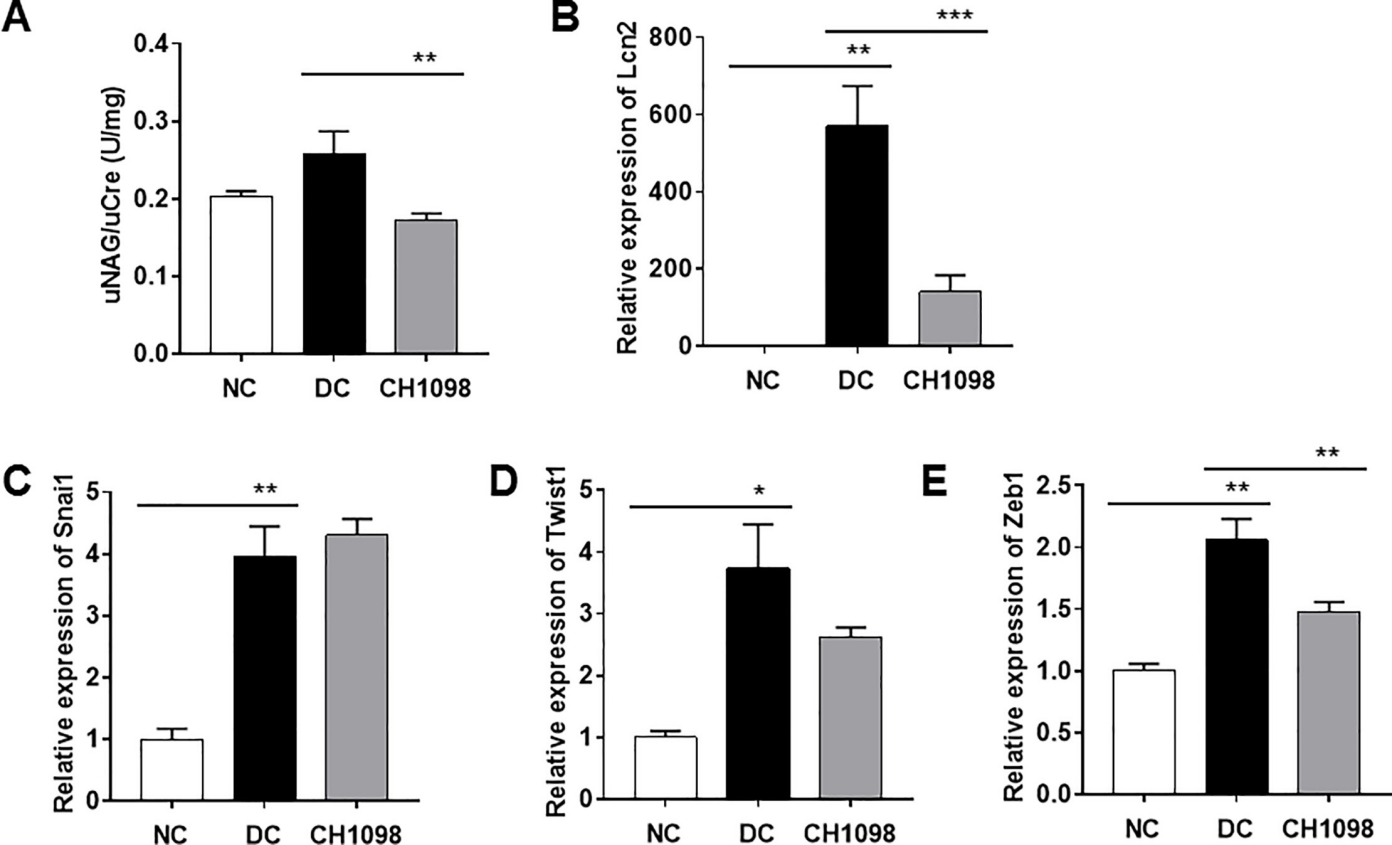
Effects of CH5451098 on tubular damage in NEP25 mouse model. CH5451098 10 mg/kg or vehicle was administered on 21 consecutive days starting one day before LMB2 injection. Urine was collected from 0 to 24 hours and kidney cortex at 24 hours after final administration of CH5451098 or vehicle, and measured for urinary NAG excretion (A), LCN2 mRNA (B), Snai1 mRNA (C), Twist1 mRNA (D), and Zeb1 mRNA (E). mRNA expression levels of each were expressed as the fold changes of NC following normalization by Mrpl19 mRNA. Concentration of uNAG was normalized against creatinine in urine. Data are expressed as mean ± SE: n = 4 in NC, n = 7 in DC, n = 10 in CH5451098 treatment *p<0.05, **p<0.01, ***p<0.001, significant difference between NC and DC, or DC and CH5451098 treatment with Student’s t-test. NC, normal control; DC, disease control; CH1098, CH5451098.

We also evaluated the effect of CH5451098 on renal tubular cells. Urinary NAG excretion was more increased in diseased control at the end of the study than in normal control (Fig 6A). This increase was significantly inhibited by CH5451098. Renal lipocalin 2 (LCN2) mRNA, a sensitive marker for tubular damage, was also significantly decreased in CH5451098 group, compared with the disease control (Fig 6B). Snai1, Twist1, and, Zeb1, which are known as EMT markers, are known to be well correlated with kidney dysfunction [32]. The expression in Zeb1 mRNA was suppressed and Twist1 was tended to suppress in CH5451098 group as compared with the disease control (Fig 6D and 6E). CH5451098 did not reduce Snai1 mRNA. Because three out of ten mice died from severe renal damage in the disease control group, the effect of CH5451098 might be underestimated. We also confirmed that the protein levels of Zeb1 and beta-catenin were reduced by CH5451098 compared to disease control (S4 Fig).

### Effects of AXL signaling on EMT in NRK-52E cells

**Fig 7:**
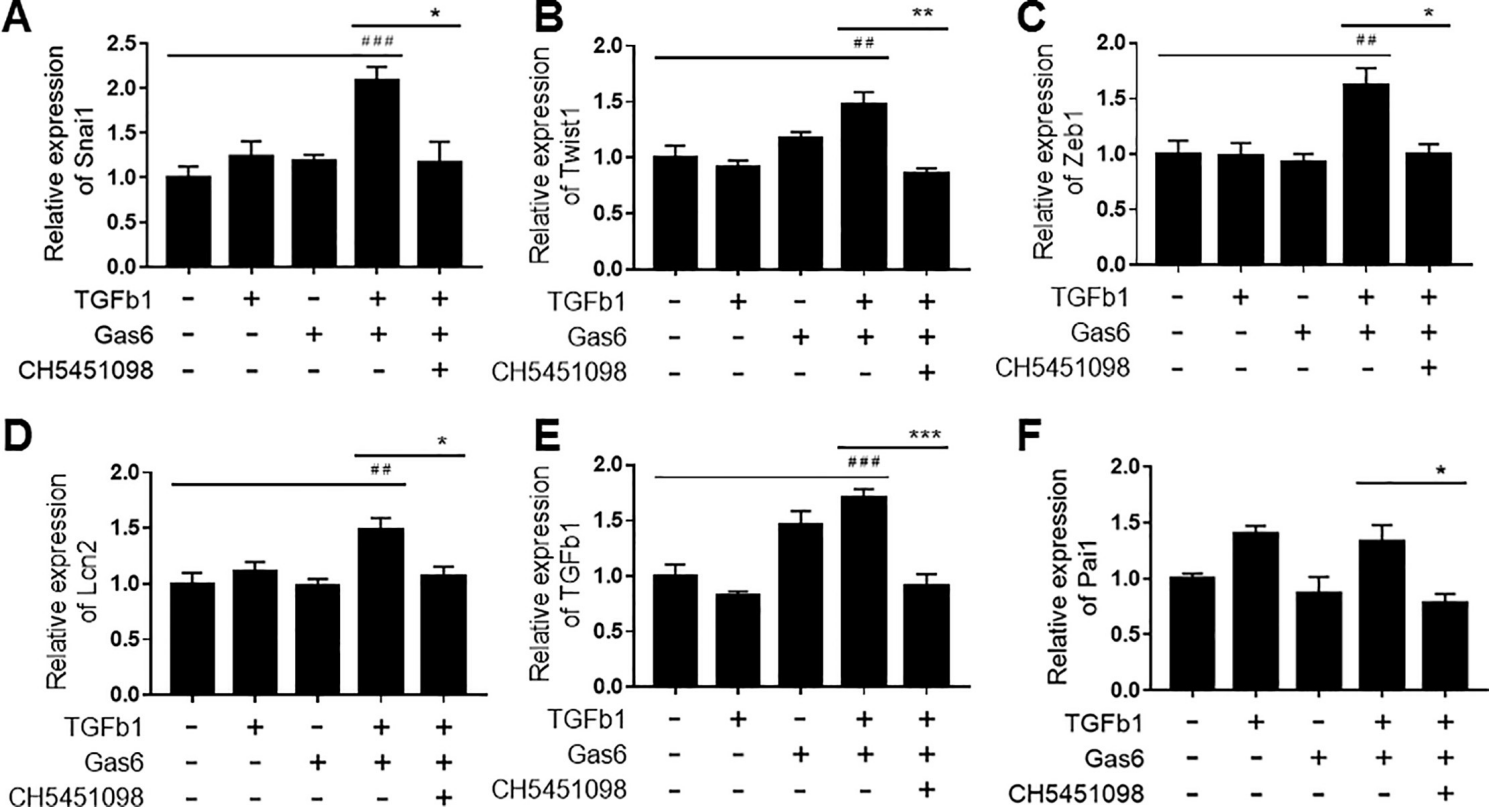
Effects of CH5451098 on tubular EMT mediated by synergistic effects of TGFβ1 and Gas6 in NRK-52E cells. A-F, mRNA expression levels of each were expressed as the fold changes of cells treated with neither TGFβ1 nor Gas6 following normalization by GAPDH mRNA. Data are expressed as mean ± SE (n = 4). Snai1 mRNA (A), Twist1 mRNA (B), Zeb1 mRNA (C), LCN2 mRNA (D), TGFβ1 mRNA (E), and Pai1 mRNA (F) #p<0.05, ##p<0.01, ###p<0.001, significant difference between cells without any stimulants with Dunnett’s t-test. *p<0.05, **p<0.01, ***p<0.001, significant difference between the cells with Gas6 and TGFβ1 stimulation and the cells with Gas6, TGFβ1 stimulation, and CH5451098 treatment with Student’s t-test.

We speculated that CH5451098 worked by inhibiting EMT in tubular epithelial cells. To support our hypothesis we evaluated the effect of CH5451098 in NRK-52E cells derived from renal tubular epithelial cells [33]. Gas6 or TGFβ1 alone were not sufficient to induce EMT markers Snai1, Twist1, and Zeb1, but Gas6 along with TGFβ1 strongly induced those expression markers. They were markedly inhibited by the AXL inhibitor (Fig 7A–7C). CH5451098 treatment inhibited not only EMT markers but also LCN2 mRNA induced by Gas6 and TGFβ1 (Fig 7D). Finally, CH5451098 also suppressed the enhancement of TGFβ1 and Pai1, similar to in vivo study (Fig 7E and 7F).

## Discussion

Previous studies have shown the controversial effects of Gas6/AXL in several kidney disease models, e.g., anti-Thy-1-induced nephritis, anti-glomerular basement membrane (GBM)-induced nephritis, streptozotocin-induced diabetic nephropathy, and renal ischemia-reperfusion [21–24, 34–37]. It is difficult to clarify the effects of Gas6/AXL signaling with AXL-KO mice because Mer that is implicated to suppress kidney dysfunction activation in the kidneys is lower in AXL-KO nephritic mice than in wild-type nephritic mice [38]. Therefore, in this study we used the potent and selective AXL inhibitor CH5451098 to elucidate the role of Gas6/AXL in kidney diseases. CH5451098 represented the suppression of disease progression in an NEP25 mouse model engineered to induce podocyte damage initially following glomeruloscrelosis, leading to interstitial fibrosis and impaired kidney function. Our results using this model which shares the features of the clinical course of glomerulonephritis reveal the importance of AXL in the pathogenesis of renal dysfunction.

Reports show that increased expression of Gas6 and AXL parallels the degree of glomerular proliferation in anti-Thy1 and anti-GBM mouse models [21, 39]. Gas6 has also been reported to induce glomerular proliferation [21, 40]. In addition to glomerular damage, tubuloinsterstitial fibrosis—showing an even stronger correlation—is also a key cause of kidney dysfunction [41]. AXL is reported to be highly expressed not only in mesangial cells but also in tubular epithelial cells from patients with IgA nephropathy or lupus nephritis, however the function of AXL in damaged tubular epithelial cells is not well understood [10]. Glomerular damage is initiated by podocytopathy and uAlb excretion is plateaued for 1–2 weeks after disease induction in NEP25 mouse model (data not shown). By the end of the study, initial glomerular damage lead to the tubular damage in this model [26]. Although renal AXL mRNA expression gradually increased over the course of the study in NEP25 mice.

Podocytes, responsive cells that block blood albumin leakage into urine, are severely damaged by the toxin used to induce kidney damage in this model [26], making it difficult to demonstrate the reduction of uAlb with drug treatment. However, such a reduction was clearly observed with CH5451098 at 3 weeks after disease induction (Fig 3C). We also found that CH5451098 has no effect on uAlb leakage at 2 weeks after disease induction compared to DC group (data not shown). We hypothesized that this was because the protection against tubular damage and renal fibrosis ultimately prevented pericapillary injury. Consequently, CH5451098 treatment attenuated not only tubular injury but also glomerular sclerosis. These responses were also supported by the histological analysis (Fig 4). These observations suggest that CH5451098 targets tubular cells rather than glomerular cells. In the histological analysis, it was obvious that CH5451098 had a strong anti-fibrotic effect. We confirmed the anti-fibrotic effect with kidney OH-Pro or collagen 1a1 mRNA analysis. These fibrotic markers were clearly inhibited with CH5451098 treatment (Fig 5A and 5B). Tubulointerstitial fibrosis, the final common pathway of glomeruronephritis, is known to be induced by tubular damage. Thus, we analyzed the effects of CH5451098 on tubular damage markers in the NEP25 mice. These markers, urinary NAG excretion and renal LCN2 mRNA, were significantly suppressed by the CH5451098 treatment (Fig 6A and 6B). TGFβ1 is known as a key factor in the progression of fibrosis in several organs [42]. Expression of TGFβ1 is greatly increased in fibrotic conditions, causing EMT of various epithelial cells. The up-regulation of AXL causes the phosphorylation of Smad3, which is regulated by TGFβ signaling and is well correlated with an increase in the metastasis of cancer [43–46]. However it remains controversial whether AXL signaling is a positive regulator of EMT [47]. In this study we have shown that AXL activation is indispensable for TGFβ1 to promote EMT in NRK-52E renal tubular epithelial cells (Fig 7A–7C). CH5451098 suppressed not only EMT markers but also TGFβ1 and its signaling (Fig 7E and 7F). TGFβ1 is reported to induce EMT through Akt activation [48]. AXL is also reported to activate Akt signaling [23]. Thus, the activation of AXL by Gas6 might enhance TGFb1-induced signal transduction to cause EMT under our experimental conditions. Phosphorylated AXL is presented at proximal tubular cells in the podocyte injury mouse model [49]. Little is known about the results of AXL phosphorylation. Our observations reveal the pathogenic role of AXL signaling through EMT in kidney dysfunction. The communication between transitioned tubular epithelial cells that modify paracrine factors and fibroblasts are reported to contribute to fibroblast proliferation and myofibroblast differentiation leading to organ fibrosis [50–52].

Gas6 and TAM receptors possess various functions in the body. Gas6 is mostly known as a coagulation factor in blood with an important role in platelet aggregation [53], but it is possible that antithrombotic activity of Gas6/AXL pathway inhibition contributes to the beneficial effects in NEP25 mouse model. We showed that AXL signaling induces EMT of tubular epithelial cells and causes tubulointerstitial fibrosis, so the inhibition of Gas6/AXL may not only reduce coagulation or glomerular damage, but can also protect tubular cells. Recently, Mer and Tyro3 have been reported to suppress kidney dysfunction [38, 54], which means the blockade of AXL and induction of Mer and Tyro3 signaling might be a promising way to cure the kidney diseases in future. In conclusion, CH5451098 is a highly selective and potent AXL inhibitor that ameliorated kidney dysfunction in glomerular nephritis through the inhibition of EMT. These results reveal that AXL strongly contributes to the disease progression of glomerular nephritis.

## Supporting information

S1 Fig Chronological change of sCre in NEP25 mice. Blood was collected at 1, 2, and 3 weeks after disease induction with LMB2. Data are expressed as mean ± SE: n = 3 in NC, n = 10 at 1 and 2 weeks, n = 8 at 3 weeks after disease induction, *p<0.05, **p<0.01, ***p<0.001, significant difference from NC with Dunnett’s t-test. NC, normal control; NEP25, disease model.
(TIF)

S2 Fig Chronological change in renal AXL mRNA in NEP25 mice. Kidney cortex was collected at 1 week, 2 weeks and 3 weeks after disease induction with LMB2. Each mRNA expression levels were expressed as the fold changes of NC following normalization by Mrpl19 mRNA. Data are expressed as mean ± SE: n = 3 in NC, n = 10 in 1 week and 2 weeks, n = 8 in 3weeks after disease induction, *p<0.05, **p<0.01, ***p<0.001, significant difference from NC with Dunnett’s t-test. NC, normal control; NEP25, disease model.
(TIF)

S3 Fig Mer and Tyro3 mRNA expression in NEP25 mice. Kidney cortex was collected at 3 weeks after disease induction with LMB2. mRNA expression levels of each were expressed as the fold changes of NC following normalized by Mrpl19 mRNA. Data are expressed as mean ± SE: n = 3 in NC, n = 7 in DC, *p<0.05, **p<0.01, ***p<0.001, significant difference between NC and DC with Student’s t-test. NC, normal control; NEP25, disease control.
(TIF)

S4 Fig ZEB1 and beta-catenin protein levels in NEP25 mice. Kidney cortex was collected at 3 weeks after disease induction with LMB2. Expression of ZEB1, beta-catenin and GAPDH in the kidney cortex were detected by western blotting. NC, normal control; DC, disease control; CH1098, CH5451098.
(TIF)

S1 File (DOCX)
