# Supplementary figures and images for: Novel AXL-specific inhibitor ameliorates kidney dysfunction through the inhibition of epithelial-to-mesenchymal transition of renal tubular cells
Source: PLoS One. 2020 Apr 23;15(4):e0232055. doi: 10.1371/journal.pone.0232055 (PMC7179907; doi:10.1371/journal.pone.0232055)

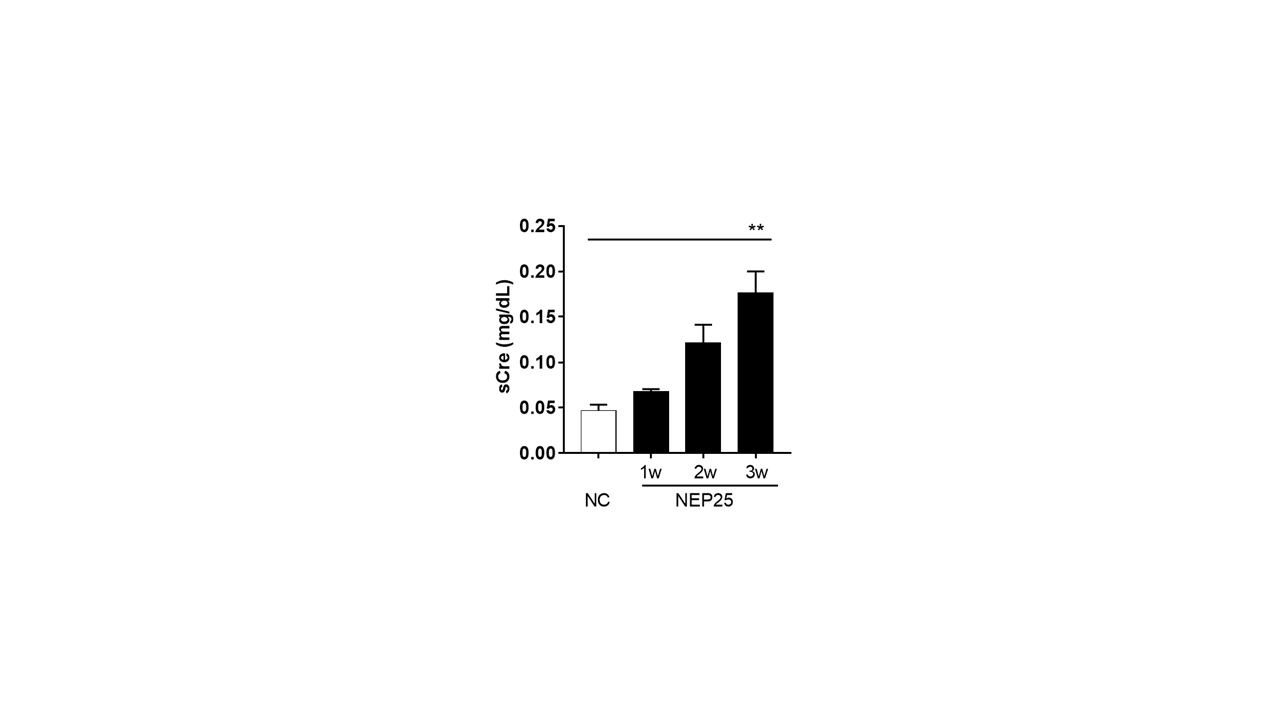

Supplement: S1 Fig — Blood was collected at 1, 2, and 3 weeks after disease induction with LMB2. Data are expressed as mean ± SE: n = 3 in NC, n = 10 at 1 and 2 weeks, n = 8 at 3 weeks after disease induction, *p<0.05, **p<0.01, ***p<0.001, significant difference from NC with Dunnett’s t-test. NC, normal control; NEP25, disease model. (TIF) [file pone.0232055.s001.tif]

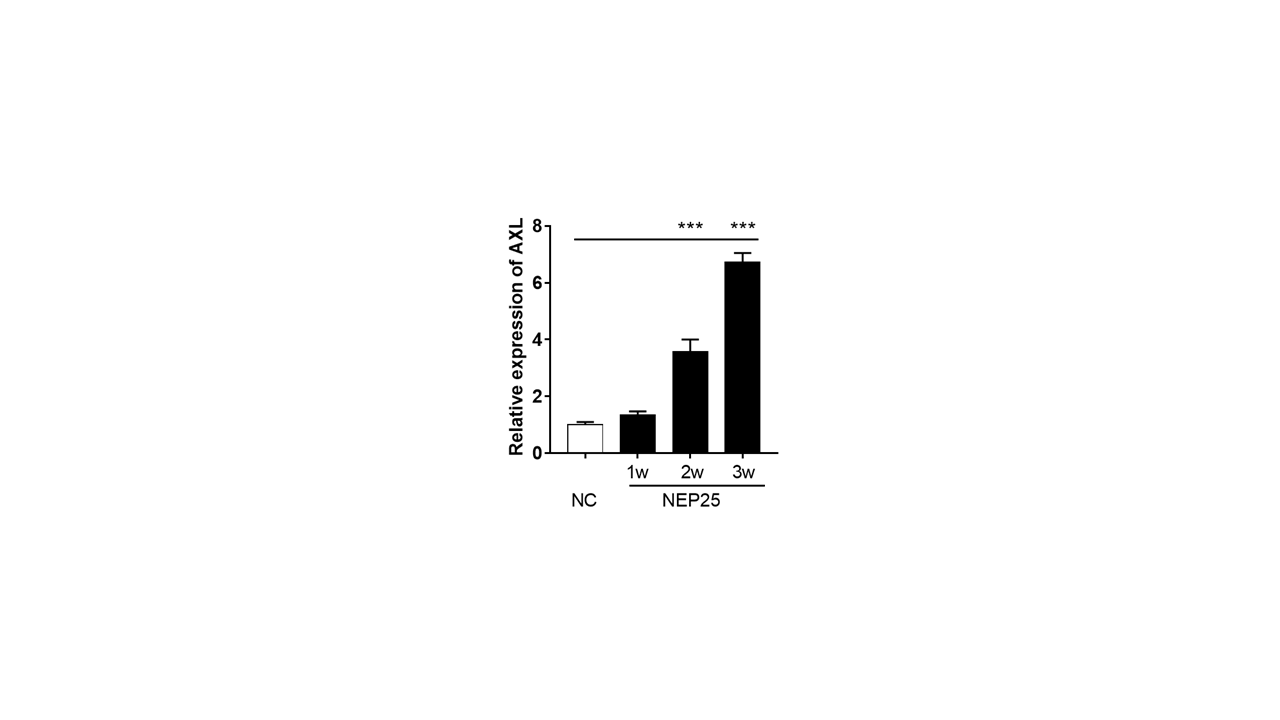

Supplement: S2 Fig — Kidney cortex was collected at 1 week, 2 weeks and 3 weeks after disease induction with LMB2. Each mRNA expression levels were expressed as the fold changes of NC following normalization by Mrpl19 mRNA. Data are expressed as mean ± SE: n = 3 in NC, n = 10 in 1 week and 2 weeks, n = 8 in 3weeks after disease induction, *p<0.05, **p<0.01, ***p<0.001, significant difference from NC with Dunnett’s t-test. NC, normal control; NEP25, disease model. (TIF) [file pone.0232055.s002.tif]

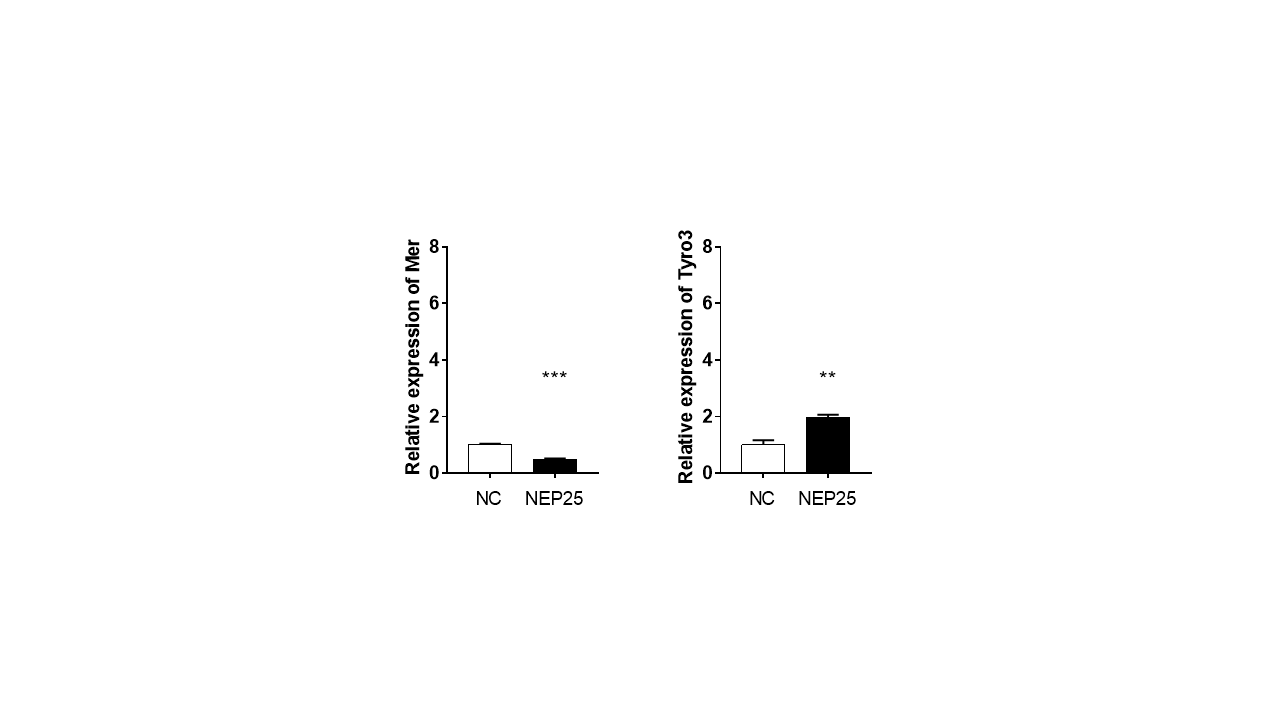

Supplement: S3 Fig — Kidney cortex was collected at 3 weeks after disease induction with LMB2. mRNA expression levels of each were expressed as the fold changes of NC following normalized by Mrpl19 mRNA. Data are expressed as mean ± SE: n = 3 in NC, n = 7 in DC, *p<0.05, **p<0.01, ***p<0.001, significant difference between NC and DC with Student’s t-test. NC, normal control; NEP25, disease control. (TIF) [file pone.0232055.s003.tif]

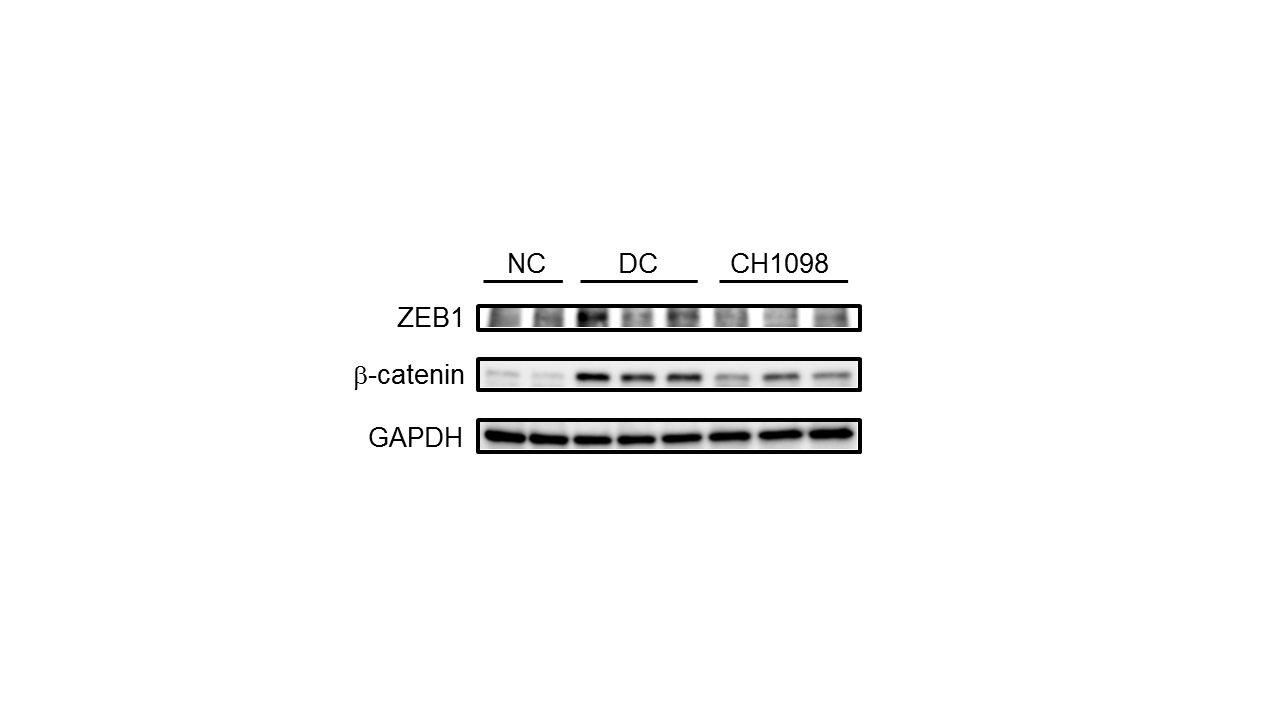

Supplement: S4 Fig — Kidney cortex was collected at 3 weeks after disease induction with LMB2. Expression of ZEB1, beta-catenin and GAPDH in the kidney cortex were detected by western blotting. NC, normal control; DC, disease control; CH1098, CH5451098. (TIF) [file pone.0232055.s004.tif]
