# Supplementary material for: Novel AXL-specific inhibitor ameliorates kidney dysfunction through the inhibition of epithelial-to-mesenchymal transition of renal tubular cells
Source: PLoS One. 2020 Apr 23;15(4):e0232055. doi: 10.1371/journal.pone.0232055 (PMC7179907; doi:10.1371/journal.pone.0232055)

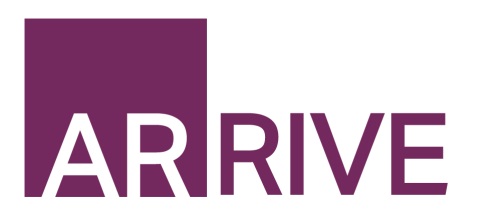


The ARRIVE Guidelines Checklist

Animal Research: Reporting In Vivo Experiments

Carol Kilkenny^1^, William J Browne^2^, Innes C Cuthill^3^, Michael Emerson^4^ and Douglas G Altman^5^

*^1^The National Centre for the Replacement, Refinement and Reduction of Animals in Research, London, UK, ^2^School of Veterinary Science, University of Bristol, Bristol, UK, ^3^School of Biological Sciences, University of Bristol, Bristol, UK, ^4^National Heart and Lung Institute, Imperial College London, UK, ^5^Centre for Statistics in Medicine, University of Oxford, Oxford, UK.*

|  | | ITEM | RECOMMENDATION | Section/ Paragraph |
| --- | --- | --- | --- | --- |
| 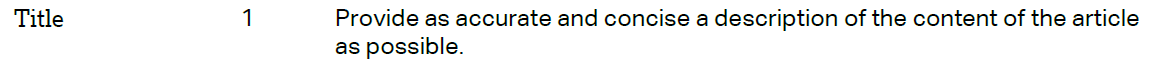 | | | Title |  |
| 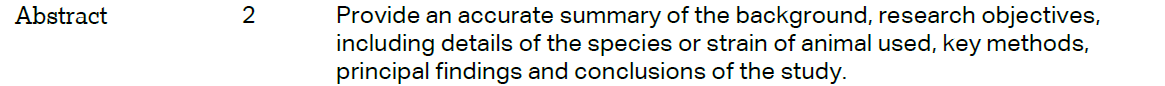 | | | Abstract |  |
| INTRODUCTION | | |  |  |
| 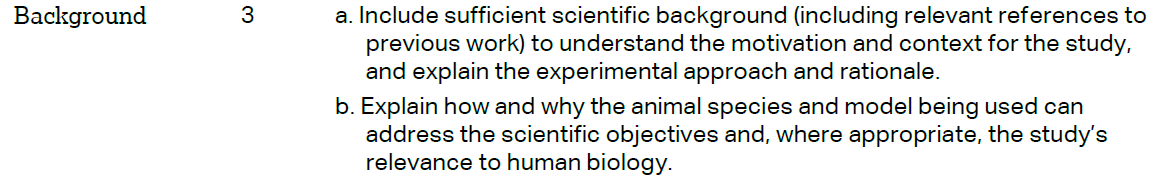 | | | Paragraphs  1-4 |  |
| 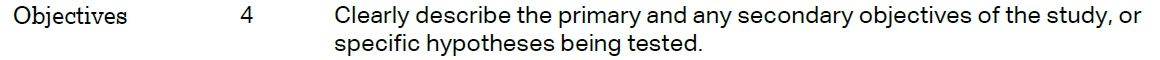 | | | Paragraph 5 |  |
| METHODS | | |  |  |
| 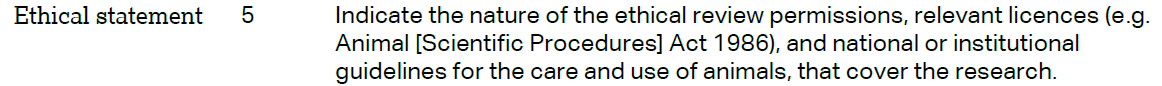 | | | “Animals” section |  |
| 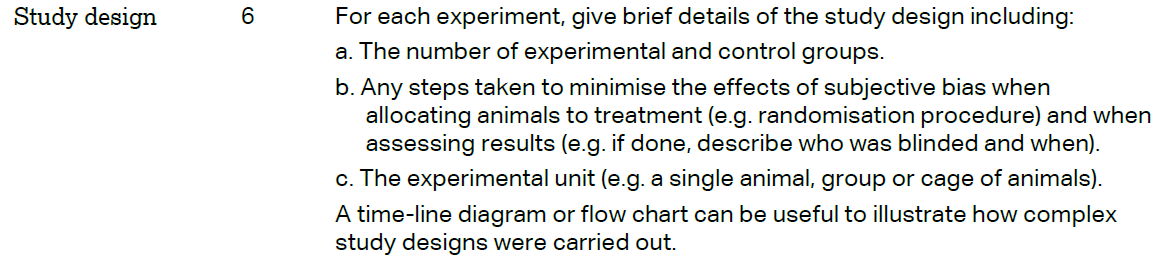 | | | “In vivo experimental design”  section |  |
| 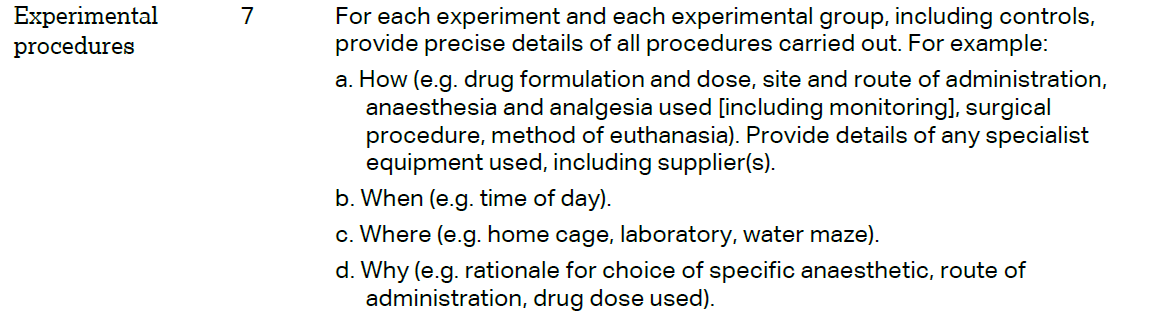 | | | “In vivo experimental design”  section |  |
| 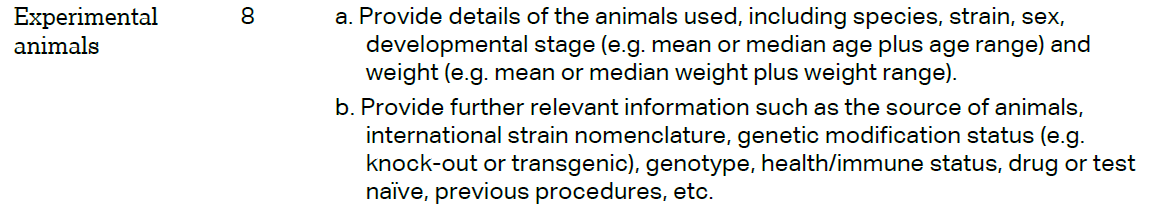 | | | “Animals” section |  |

The ARRIVE guidelines. Originally published in *PLoS Biology*, June 2010^1^

| 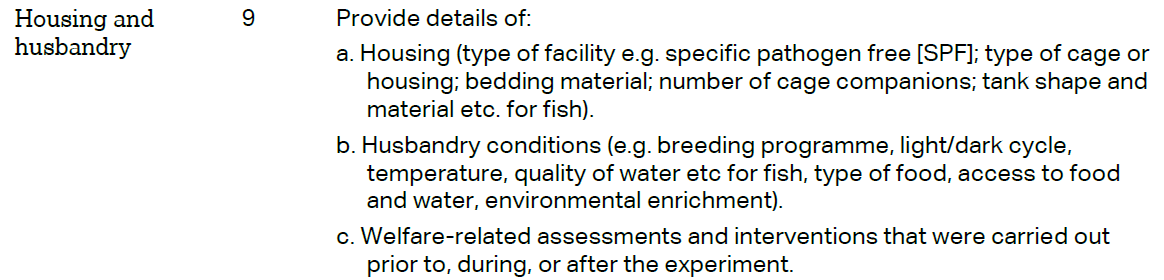 | “Animals” section | |
| --- | --- | --- |
| 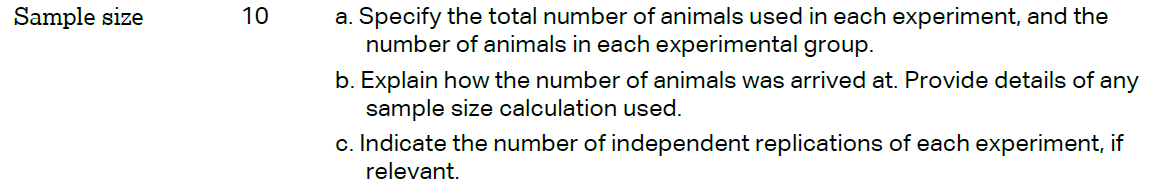 | “In vivo experimental design”  section | |
| 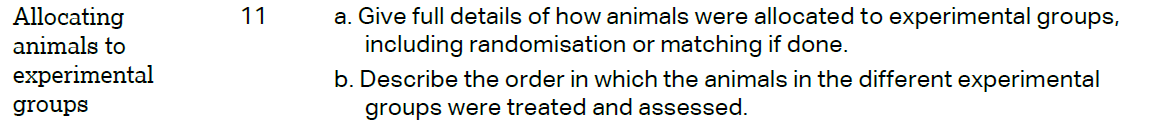 | “In vivo experimental design”  section | |
| 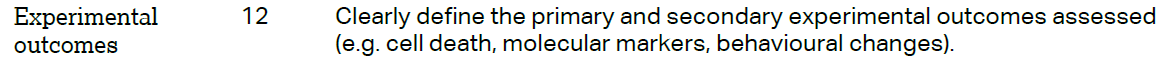 | Biochemical analysis, Gene expression analysis, Western blotting, Collagen analysis, Pathological analysis | |
| 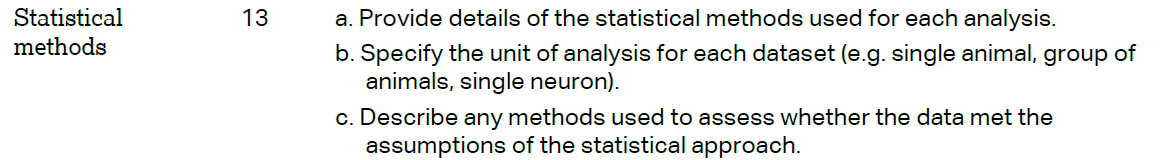 | “Statistical analysis” section | |
| RESULTS |  | |
| 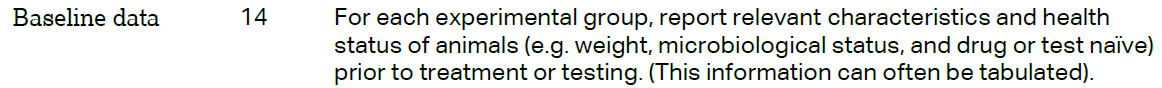 |  | |
| 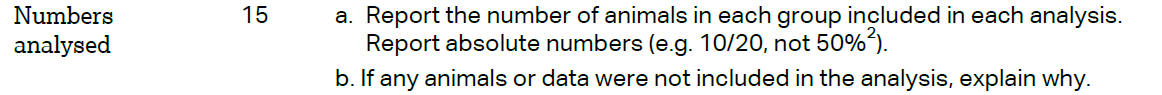 | Paragraphs 2-4 | |
| 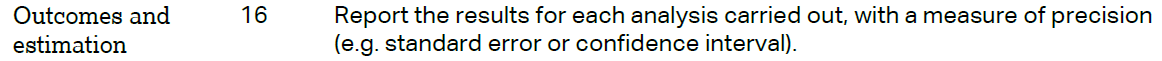 | Paragraphs 2-4 | |
| 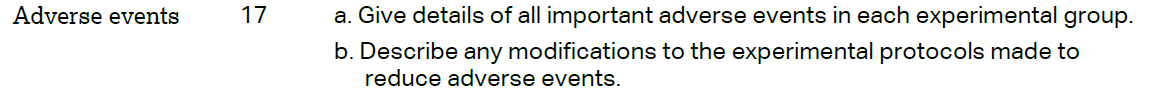 | No adverse events without renal damage | |
| DISCUSSION |  | |
| 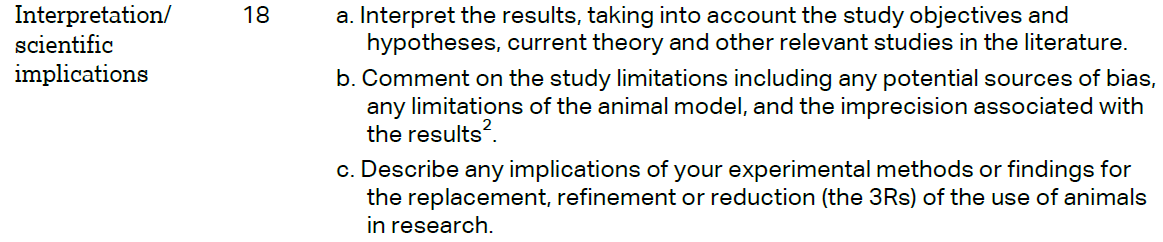 | Throughout the discussion section | |
| 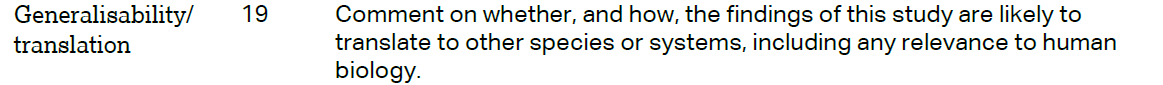 |  | |
| 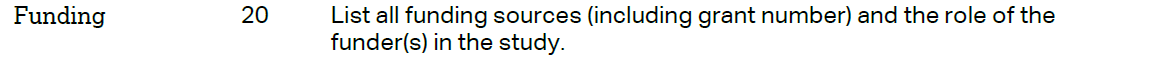 | |  |


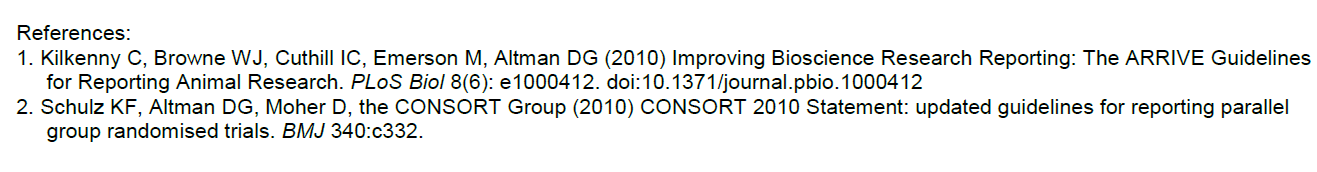

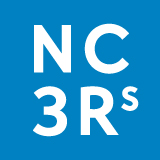

Supplement: S1 File — (DOCX) [file pone.0232055.s005.docx]
